# Unveiling the nonlinear dynamics of player performance in China’s super league as a function of age

**DOI:** 10.1038/s41598-024-65766-y

**Published:** 2024-07-09

**Authors:** Jun Cao, Wei Zhang, Changjing Zhou

**Affiliations:** School of Athletic Performance, Shanghai University of Sport, Shanghai, China

**Keywords:** Football, Performance, Age, Generalized additive model, Nonlinearity, Physiology, Psychology

## Abstract

To explore the dynamics in physical and technical performance of professional football players and changes across age groups. Match statistics were collected from 1900 games across ten seasons (2012–2021) in the Chinese Super League. Generalized additive models visualized age-related trends in 12 key performance indicators including technical and physical variables. Revealed nonlinear trajectories characterized by rapid early declines, stable peak periods and accelerated late decreases. Physical indicators decreased progressively from the early 20 s before stabilizing briefly then declining further after 30. Conversely, technical metrics gradually improved into the late 20 s and early 30 s prior to decreasing again. This study provides novel evidence that football performance changes nonlinearly across age. Targeted training and development strategies should be tailored to the specific needs of different career stages.

## Introduction

The aging process plays a critical role in determining the competitive performance of football players across their sporting career^1^. Understanding how match-related physical, technical and tactical capabilities change across age provides valuable insights to guide player development and team management^2^. However, previous studies have mostly described generalized linear declines assuming players progressively worsen year after year. This fails to capture potential nonlinear patterns and nuanced differences between career stages^3^. Recent research suggests growth, stabilization and decrement phases may better characterize lifespan trajectories in diverse domains including cognitive and physical abilities^4,5^. Exploring football performance changes using models allowing for nonlinear patterns remains limited.

Professional football teams comprise athletes across a wide age range^6^. The average career duration is approximately 8–11 years generally ending between ages 31–35^7^. Prior studies identified peak performance occurring around 25–27 years in top European leagues^3^. Many clubs hold beliefs that players decline significantly after age 30, leading to shorter contracts for veterans despite limited empirical evidence^3^. While physical capacities inevitably decline from peak ages, technical skills may remain robust into the 30s^8^. Understanding intricacies in age-related performance changes provides an opportunity to optimize player development, training and team selection across different career stages. However, existing football research predominantly utilizes cross-sectional designs confounded by historical and selection biases^9^. Even longitudinal studies generally apply linear statistical models unable to capture potential nonlinear changes aligned with maturation processes^10^.

Generalized additive models (GAMs) provide effective modeling of nonlinear patterns^11^. GAMs make no assumptions about underlying trends, enabling evaluating potential nonlinear changes in performance across ages^12^. Therefore, this study aimed to analyze age-related changes in the physical and technical performance of professional football players competing in the Chinese Super League (CSL) using generalized additive models. It was hypothesized that the trajectories would demonstrate nonlinear patterns characterized by more precipitous declines in the early and later career stages interspersed with a period of stabilization around the peak ages.

## Materials and methods

### Sample

The study included information about outfield players from the first professional men’s soccer league of China (Chinese Super League, CSL). Data included the actions of a total of 1346 native players in 2400 matches during the 2012–2021 seasons (ten seasons total). Due to the emergence of red cards, the decrease in the player number of one team will cause the imbalance of the game. Therefore, in order to differentiate balanced matches and unbalanced matches, firstly we ruled out the matches where there emerged red card(s) during the course of the match, leaving 1990 matches selected. The unit of analysis was individual player match observations. The final sample consisted of 32,348 observations. And in order to provide a clearer representation of the sample, we conducted a statistical analysis of the number of players in different age groups, as shown in Table 1.

**Table 1:** The sample size for different seasons and age groups, as well as the total sample size for 10 seasons and different age groups.

Season	Grop 1 (17–22.9 years)	Grop 2 (23–28.9 years )	Grop 3 (29–34.9 years )	Grop 4 (35–40.9 years)	Grop 4 (41–44.9 years)
2012	503	2010	990	14	0
2013	283	2225	898	25	0
2014	326	1968	895	154	0
2015	342	2035	1056	110	0
2016	115	1971	1302	172	0
2017	334	2205	1224	178	0
2018	294	1546	1914	186	0
2019	333	1539	1782	116	7
2020	179	1109	1186	155	0
2021	61	285	283	33	1
Total	2770	16,893	11,530	1143	8

### Data collection

Physical and technical performance data were provided by the Amisco tracking system (Amisco, Nice, France). The validity and reliability of this system have been verified for quantifying player metrics^13^. Raw data were extracted and imported into a customized database. Only outfield players participating in over 50% of league matches were included, consistent with previous studies^14^. Players’ match data were normalized to account for substitutions and game duration using the formula:

$$ {\text{V}}_{{{\text{standardized}}}} \, = \,\left( {{\text{V}}_{{{\text{original}}}} {\text{/Time}}\,{\text{played}}} \right) \times \left( {{\text{Full}}\,{\text{match}}\,{\text{time}}} \right) \times {1}00\% . $$

Standardization provided more consistent match profiles^15^. Based on literature precedents^16–18^, six technical indicators and six physical indicators representing key elements of football performance were selected for analysis as defined in Table 2.

**Table 2:** Operational definition of technical and physical performance-related parameters.

Physical performance-related parameters: operational definition
(TD) Total distance (km): distance covered in a match by all the players of a team
(LSR) Low-speed-running (m): distance covered at a speed of 7.1→14.3 km/h in a match
(MSR) Middle-speed-running (m): distance covered at a speed of 14.3→19.7 km/h in a match
(HSR) High-speed-running (m): distance covered at a speed of 19.7→25.1 km/h in a match
Sprint (m): distance covered at a speed over 25.1→ + km/h in a match
(NS) Number of sprints (n): number of sprints covered at the speed over 25.1→ + km/h in a match
Technical Performance-Related Parameters: Operational Definition
Pass (n): an intentional played ball from one player to another
Pass accuracy (%): successful passes as a proportion of total passes
(NC) Number of challenge(n): actions when two players are competing for ball possession, which is not in the control of any player, i.e., both players have approximately a 50% chance of gaining control of the ball; includes ground and air challenges
Challenge accuracy (%): successful challenges as a proportion of the total challenges
Ball retention (%): ball retention is defined as being when your team keeps possession of the ball following your pass, which can be complete or incomplete
(FP)First time Pass of individual possession (%): The percentage of immediate passes made by a player after gaining possession out of the total times the player gained possession

### Statistical analysis

Data were analyzed using RStudio (Version 4.0.2) with R software (Version 4.3.0.). Generalized additive models (GAMs) were constructed using the package gamm4 to flexibly model nonlinear patterns in the performance variables across age. GAMs avoid imposing predefined functional forms and estimate smooth regression curves from the data^11^. To account for individual differences and correlations between repeated measures data across ages for the same player, players were included as a random effect in the models. Physical and technical indicators were modeled separately as the dependent variables, with age as the independent variable. Gam plots visualized estimated smooth functions showing the age-related changes. Statistical significance was defined as p < 0.05.

## Results

**Figure 1:**
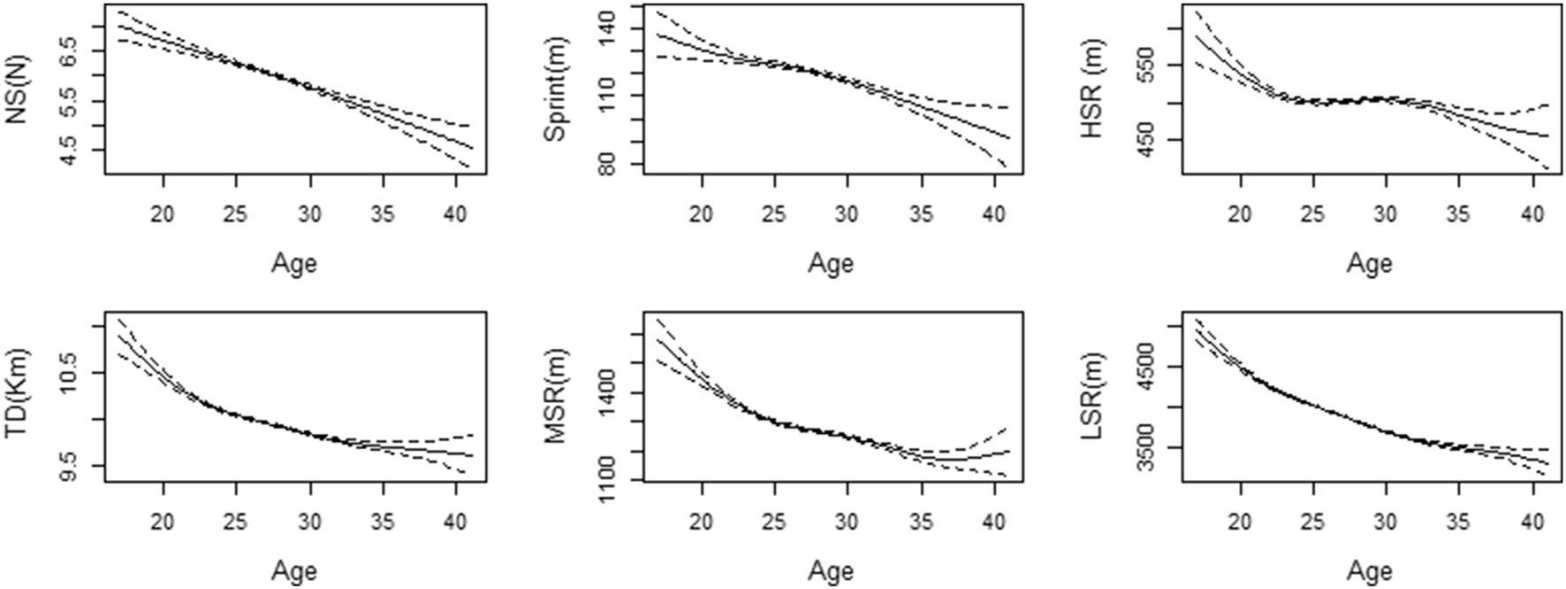
Generalized additive model plots for age-related changes in physical performance metrics. Solid lines indicate smoothers surrounded by 95% confidence intervals depicted by dashed lines.

**Figure 2:**
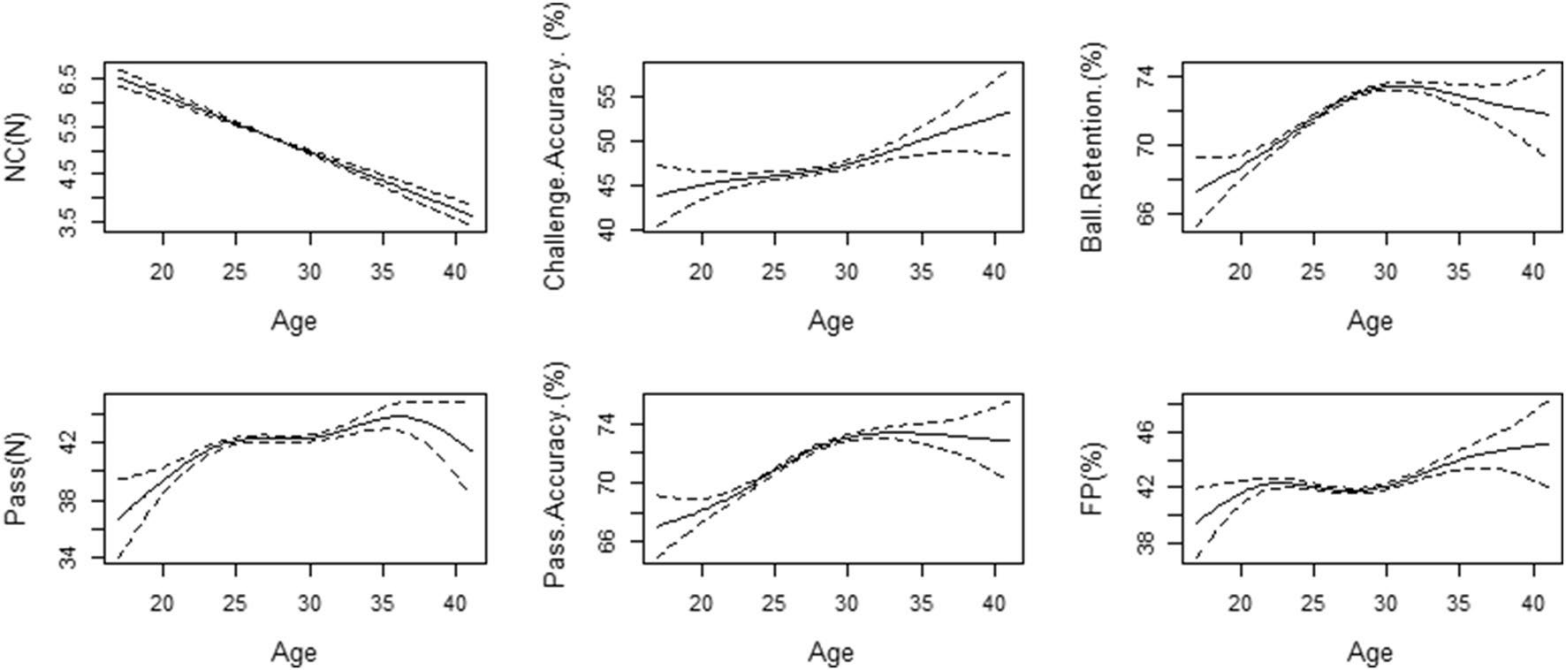
Generalized additive model plots for age-related changes in technical performance metrics. Solid lines indicate smoothers surrounded by 95% confidence intervals depicted by dashed lines.

Figures 1 and 2 illustrate distinct performance indicators, namely number challenge (R^2^ = 0.398; *P* < 0.001) and number sprint (R^2^ = 0.526; *P* < 0.001), both of which exhibit a consistent decline as individuals progress in age. Notably, a discernible trend of diminishing performance is evident in sprint (R^2^ = 0.515; *P* < 0.001) and high speed running (R^2^ = 0.699; *P* < 0.001) indicators as age advances, with the rate of decline exhibiting notable variability across different age cohorts. This decline, however, presents intriguing nuances: between the ages of 23 and 28, the rate of decline notably decelerates, and in the case of high speed running, an intriguing stabilizing trend emerges. Nevertheless, both indicators experience rapid declines prior to the age of 23 and beyond the age of 30. Similarly, TD (R^2^ = 0.875; *P* < 0.001), medium speed running (R^2^ = 0.779; *P* < 0.001), and low speed running (R^2^ = 0.857; *P* < 0.001) display coherent age-related patterns. These indicators also exhibit an accelerated decrease before the age of 23, followed by a gradual attenuation in the rate of decline after this age threshold. Notably, medium speed running is a noteworthy exception, revealing a slight increase after the age of 35.

The performance indicators pass (R^2^ = 0.471; *P* < 0.001) and challenge accuracy (R^2^ = 0.172; *P* < 0.001) both exhibit an upward trajectory prior to the age of 25. Notably, Challenge accuracy displays a gradual increase, while pass demonstrates a more pronounced improvement. Following the age of 25, challenge accuracy demonstrates a consistent linear growth, while Pass maintains a steady performance from ages 23–28, subsequently experiencing progressive enhancement until a rapid decline becomes evident around the age of 35. Moreover, pass accuracy (R^2^ = 0.262; *P* < 0.001) and ball retention (R^2^ = 0.219; *P* < 0.001) both exhibit an ascending pattern before the age of 30. Pass accuracy undergoes a rapid surge, whereas ball retention displays linear growth. Beyond the age of 30, ball retention gradually decreases, while pass accuracy displays distinct trends across various age groups. Specifically, it tends to stabilize between the ages of 30 and 35 before resuming a gradual rise. Lastly, the indicator FP (R^2^ = 0.305; *P* < 0.001) demonstrates rapid escalation with age until the age of 23, after which the growth trend diminishes and approaches stability between ages 23 and 28. Following the age of 30, FP once again embarks on a progressive upward trend.

## Discussion

This study analyzed age-related changes in the match performance of professional football players competing in the CSL across ten seasons. Generalized additive models revealed nonlinear trajectories characterized by more rapid declines in the early career and later veteran stages interspersed with periods of stabilization around the peak ages.

Physical capacities are crucial determinants of football performance, underpinning actions like repeated sprinting, jumping and changes of direction^19^. However, physical capabilities eventually decline due to age-related changes in muscle morphology and physiology^20^. Consistent with prior research^8^, our results exhibited decreasing total distances covered as players progressed through their 20 s. High-speed activities showed pronounced drops early in careers, aligning with studies demonstrating running-based attributes peak around 22–23 years^21,22^. Interesting, from ~ 23 to 28 years high-speed running stabilized suggesting players adapted training to maintain athleticism^23^. Thereafter, sharp downward shifts likely indicated deteriorating physiological capacities that could not be attenuated^20^.

Conversely, most technical indicators improved into the late 20 s and early 30 s. Gradual refinement of passing, challenges and ball control over this period highlights the ongoing development of tactical skills and decision-making^18^. Cognitive abilities enable older players to offset physical losses by applying experience and pattern recognition^24^. Thereafter, downward turns probably reflected declining physical capacities constraining technical execution^8^. However, passing accuracy and challenge success continued improving suggesting players retained remnants of developed technique^3^.

Overall, these findings provide novel insights into football performance dynamics over careers. The nonlinear changes align with lifespan development models describing phases of growth, maintenance and decline^4,5^. Targeted training interventions should be tailored to athletes’ specific career stages^10^. For instance, developing physical capacities is crucial initially before shifting emphasis to technical and tactical refinement during peak ages. Thereafter maintaining fitness while applying cognitive experience becomes paramount.

Strategic player recruitment and team development policies could also leverage understanding of age-related performance trajectories^25^. Blending youthful athleticism with veterans’ tactical expertise within squads may maximize overall capabilities^26^. Furthermore, substituting players demonstrating accelerated declines for fresh legs late in games could enhance team performance^27^. Consideration of both current ability levels and projected progression rates can inform team selections and contracts.

## Conclusion

In conclusion, this study revealed age-related changes in professional football match performance followed nonlinear trajectories characterized by more precipitous declines in early and later career stages compared to during peak ages where stabilization occurred. Physical capacities decreased progressively from the early 20 s before briefly stabilizing then falling sharply again after 30 years. Conversely, technical indicators improved into the late 20 s and early 30 s before subsequent decreases. These results provide novel evidence that football performance evolves nonlinearly across the lifespan. Targeted development strategies tailored to athletes’ specific career stages should be designed leveraging these insights. Furthermore, understanding nonlinear performance changes provides an opportunity to strategically optimize player recruitment, team development and in-game decisions.

### Practical applications

The findings from this study highlight several practical implications for player development and team management in professional football.

Customizing adaptive physical conditioning for different stages of a professional career is crucial. Early career: emphasize maximizing sprinting and endurance. Peak: focus on skill development while ensuring physical health. Later career: maintain physical abilities. Recruitment should anticipate career progression rates, not just current skills. Optimizing overall team strength involves integrating promising young players with tactically experienced veterans. Substituting fresh players for fatigued ones late in games can offer a tactical advantage. Similarly, reducing playing time for aging athletes can enhance performance.

Overall, an integrated approach leveraging understanding of nonlinear performance changes across age can inform strategies enhancing player development, team balance and in-game management. Findings highlight the ongoing refinement of physical power, technical execution and tactical expertise required throughout careers in the dynamic, multifaceted sport of football.

### Limitations

This study has certain limitations. Modeling physical and technical indicators separately is not conducive to showing the mutual effects of technology and physical fitness on players in games. Only CSL matches were assessed and findings require verification across diverse leagues and levels of play. Finally, variations between player positions were not addressed. Future studies should examine larger samples over multiple seasons and leagues. Exploring differences between player positions would provide further insights.
